# Adaptive Responses to PARP Inhibition Predict Response to Olaparib and Durvalumab in the AMTEC Trial

**DOI:** 10.21203/rs.3.rs-11196611/v1

**Published:** 2026-10-23

**Authors:** Zahi I. Mitri, Jayne M. Stommel, Allison L. Creason, Tugba Y. Ozmen, Matthew J. Rames, Furkan Ozmen, Daniel Bottomly, Boyoung Jeong, Jeong Youn Lim, Shamilene Sivagnanam, Konjit Betre, Jinho Lee, Marilyne Labrie, Lisa M. Coussens, Christopher L. Corless, Shannon K. McWeeney, Gordon B. Mills

## Abstract

Metastatic triple-negative breast cancer (mTNBC) has limited therapeutic options, particularly for non-BRCA1/2-mutated (non-BRCAm) tumors, where PARPi monotherapy has limited activity. Based on strong preclinical evidence, the phase II AMTEC trial (NCT03801369) evaluated olaparib plus durvalumab in non-BRCAm mTNBC, using a one-month olaparib monotherapy lead-in with paired pre-treatment and on-olaparib biopsies for multi-omic profiling of adaptive treatment responses. The primary endpoint, overall response rate, was 29%; secondary endpoints included a clinical benefit rate of 48%, progression-free survival of 4.8 months, and overall survival of 15.5 months, with two durable complete responses. The trial closed early without meeting its primary endpoint; however, these promising responses prompted exploratory multi-omic analyses to identify robust response predictors, including a three-protein immune signature translatable to routine IHC. On-olaparib biopsies yielded more significant predictors than pre-treatment biopsies, indicating that PARPi-induced adaptive changes in tumor and immune biology contribute to benefit from combined PARPi and immune checkpoint blockade.

